# Reconfigurable Complementary Logic Circuits with Ambipolar Organic Transistors

**DOI:** 10.1038/srep35585

**Published:** 2016-10-20

**Authors:** Hocheon Yoo, Matteo Ghittorelli, Edsger C. P. Smits, Gerwin H. Gelinck, Han-Koo Lee, Fabrizio Torricelli, Jae-Joon Kim

**Affiliations:** 1 Department of Creative IT Engineering, Pohang University of Science and Technology (POSTECH), Pohang 790-784, Korea; 2 Department of Information Engineering, Università degli Studi di Brescia, via Branze 38, 25123 Brescia, Italy; 3 Holst Centre, TNO-The Dutch Organization for Applied Scientific Research, High Tech Campus 31, 5656 AE Eindhoven, The Netherlands; 4 Pohang Accelerator Laboratory, Pohang 790-784, Korea

## Abstract

Ambipolar organic electronics offer great potential for simple and low-cost fabrication of complementary logic circuits on large-area and mechanically flexible substrates. Ambipolar transistors are ideal candidates for the simple and low-cost development of complementary logic circuits since they can operate as n-type and p-type transistors. Nevertheless, the experimental demonstration of ambipolar organic complementary circuits is limited to inverters. The control of the transistor polarity is crucial for proper circuit operation. Novel gating techniques enable to control the transistor polarity but result in dramatically reduced performances. Here we show high-performance non-planar ambipolar organic transistors with electrical control of the polarity and orders of magnitude higher performances with respect to state-of-art split-gate ambipolar transistors. Electrically reconfigurable complementary logic gates based on ambipolar organic transistors are experimentally demonstrated, thus opening up new opportunities for ambipolar organic complementary electronics.

Organic and polymeric materials deposited at low cost on large-area mechanically flexible substrates are the basis for ubiquitous and imperceptible electronic surfaces integrated in smart objects, thus opening new application opportunities in several fields, including for example entertainment, wellness, security, communication, mobility, healthcare, etc^1^,^2^,^3^,^4^,^5^. The great potential offered by organic technologies can meet the application requirements in terms of robustness^6^, low-power consumption^7^, and high-functionality^8^,^9^ by adopting complementary logic circuits, which rely on the availability of both hole-channel (p-type) and electron-channel (n-type) transistors.

The fabrication of p- and n-type organic thin-film transistors (OTFTs) requires the development and deposition of two different semiconductors^10^,^11^,^12^. In addition, each of them has to be carefully optimized by suitable temperature annealing^13^ and appropriate engineering of both the insulator-semiconductor^14^,^15^,^16^ and the metal-semiconductor interfaces^17^,^18^,^19^,^20^,^21^. Typically, a proper selection of orthogonal solvents coupled with additive patterning techniques^22^, such as inkjet-printing^23^,^24^, is employed to provide patterned p- and n-regions in solution-processed devices. However, the resulting fabrication process is complex and the device density is low.

A promising alternative approach is to use ambipolar OTFTs where both electrons and holes can be injected and transported in the same ambipolar organic semiconducting layer^25^. Depending on the applied voltages, ambipolar transistors can operate as p- or n-type transistors, or as p-n junctions^26^,^27^,^28^,^29^,^30^. Novel gating techniques based on split-gate or tri-gate architectures allow to set the transistor polarity by preventing the charge injection of one of the carrier type (holes or electrons) from the drain^31^,^32^,^33^,^34^. Unfortunately, the spacing between the multiple gate electrodes (named gap) severely limits the drain current^34^. The maximum drain current obtained in ambipolar OTFTs with gaps can be up to one order of magnitude lower than that of full-gate ambipolar OTFTs fabricated in the same technology. To maximize the transistor performances the gap size has to be as small as possible. This is technologically demanding because nanometre-size gaps are difficult to obtain. In addition, a small variation of the gap size may result in a large variation of the drain current since the transistor characteristics are strongly affected by the gaps. While multiple gate transistors are a very promising approach, the aforementioned issues are currently hampering their use for the development of ambipolar complementary organic circuits.

By depositing two metal layers, one for the main gate and one for the side gate, here we show high-performance ambipolar organic transistors with electrical control of the polarity. Owing to the non-planar structure, the gap(s) between the gates are avoided and a continuous full-accumulated channel is formed. Non-planar gate ambipolar transistors show efficient operation and the maximum hole and electron drain currents, normalized by the transistor geometries, are more than one order of magnitude higher than state-of-art split-gate ambipolar transistors, thus providing inverters with superior performances. By means of two-dimensional numerical simulations we provide a comprehensive understanding of non-planar gate ambipolar transistors, shedding light on the key device parameters. Finally, reconfigurable complementary logic gates – the basic building blocks of digital integrated circuits – based on ambipolar organic transistors are experimentally demonstrated for the first time. The superior performance of non-planar split gate combined with the design optimization ensured by reconfigurable organic logic gates opens up new opportunities for the development of large area flexible circuits and smart sensors.

## Results

### Non-planar gated ambipolar organic transistor

**Figure 1:**
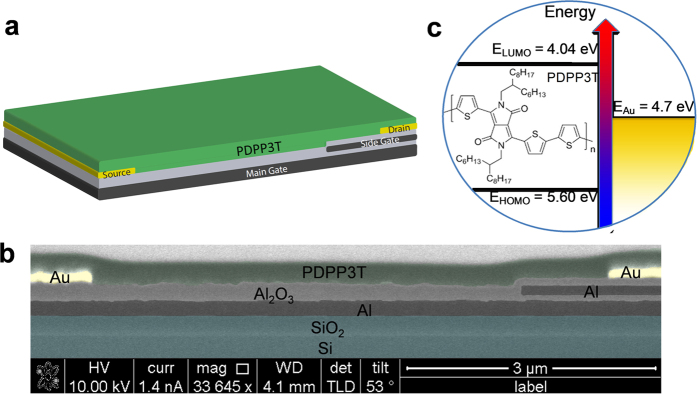
Non-planar split-gate ambipolar transistor. (**a**) Three-dimensional structure. (**b**) SEM image (**c**) Schematic band diagram of poly[{2,5-bis(2-hexyldecyl)-2,3,5,6-tetrahydro-3,6-dioxopyrrolo[3,4-c]pyrrole-1,4-diyl}- alt -{[2,2′:5′,2′′-terthiophene]-5,5′′-diyl}] (PDPP3T) in relation with gold (Au).

The simplified cross-section of the non-planar split-gate ambipolar OTFTs (NPA-OTFTs) is shown in Fig. 1a. The transistors are based on the bottom gate bottom contacts structure where an additional metal layer, named side gate, is located in between the gate and drain electrodes. The side gate is used to control the charge injection at the drain electrode. The gate and the side gate are separated along the vertical direction and not horizontally as in coplanar split-gate transistors^31^,^32^,^33^,^34^. A typical cross-section SEM image of the transistors is shown in Fig. 1b. Aluminum gate and side gate electrodes are patterned using photolithography. Thereafter we apply atomic layer deposited aluminum oxide (Al_2_O_3_), followed by Ti–Au source and drain electrodes patterned by a lift-off process. The channel length and width are L = 6 μm and W = 810 μm, respectively. The side gate overlaps with the drain electrode and it extends over the drain electrode for L_SG_ = 1 μm. The Al_2_O_3_ is treated with octadecylphosphonic acid (ODPA) and poly[{2,5-bis(2-hexyldecyl)-2,3,5,6-tetrahydro-3,6-dioxopyrrolo[3,4-c]pyrrole-1,4-diyl}- alt -{[2,2′:5′,2′′-terthiophene]-5,5′′-diyl}] (PDPP3T) is deposited by spin coating^35^,^36^. Further details are reported in the *Methods* Section. The molecular structure and the energy band diagram of the ambipolar semiconducting polymer PDPP3T are shown in Fig. 1c. The highest occupied molecular orbital (HOMO) and the lowest unoccupied molecular orbital (LUMO) levels measured by means of ultraviolet photoelectron spectroscopy are 5.60 and 4.04 eV, respectively (Supplementary Fig. 1). Gold was used for both the hole and electron injecting electrodes for practical convenience, owing to its environmental stability and its ease of patterning by photolithography.

**Figure 2:**
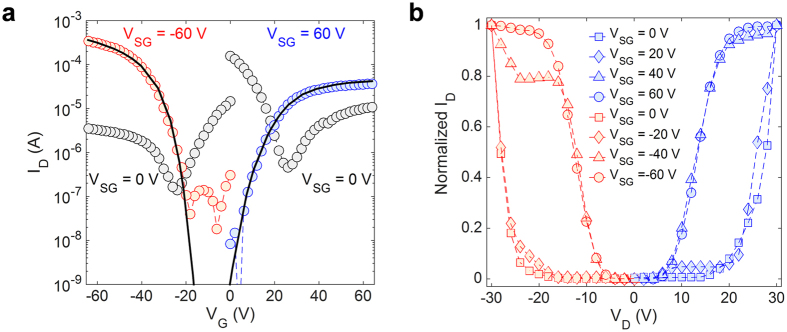
Electrical characteristics of non-planar split-gate ambipolar transistor. The transistors channel length and width are L = 6 μm and W = 810 μm, respectively. (**a**) Measured (symbols) transfer characteristics (I_D_-V_G_) at |V_D_| = 60 V, of PDPP3T operated in ambipolar V_side_ = 0 V, and unipolar p-type V_side_ = −60 or n-type V_side_ = 60 V transistor. Full lines are calculated by means of 2D numerical simulations. (**b**) Normalized output characteristics (I_D_-V_D_) as a function of the side gate voltage V_side_. |V_G_| = 20 V.

Measured transfer and output characteristics are shown in Fig. 2. Depending upon the voltage applied to the side gate V_side_, non-planar ambipolar transistors can operate as conventional ambipolar, p-type, or n-type OTFTs. Figure 2a shows that at V_side_ = 0 V both electrons and holes can be injected and typical V-shape characteristics (grey symbols) are measured. In contrast, when V_side_ = −60 V electron injection at the drain contact is prevented and the NPA-OTFTs show p-type operation (red symbols). Analogously, when V_side_ = 60 V hole injection at the drain contact is prevented and the NPA-OTFTs show n-type operation (blue symbols). The output characteristics shown in Fig. 2b confirm that the polarity of NPA-OTFTs can be set by the side gate voltage V_side_. In order to provide an easy comparison, in Fig. 2b the drain current is normalized with respect to its maximum. Typical ambipolar characteristics are measured at small |V_side_| while, at large |V_side_|, the unwanted charge injection from the drain electrode is prevented and unipolar characteristics with flat current saturation are obtained.

When the NPA-OTFTs work in unipolar regime, the on/off current ratio is about 10^4^ for both electrons and holes, the turn-on voltages are V_on,h_ ≈ −20 V and V_on,e_ ≈ 0 V, and the average transconductance for p-type and n-type operation are g_m-p_ = 1.60 μS and g_m-n_ = 0.19 μS, respectively. For comparison, we fabricated conventional (viz. single gate) ambipolar and co-planar split-gate OTFTs with 1 μm gate-gap in the same technology (Supplementary Fig. 3). The average transconductance obtained in conventional ambipolar OTFTs are g_m-p_ = 1.40 μS and g_m-n_ = 0.16 μS, while in coplanar split-gate OTFTs are g_m-p_ = 0.40 μS and g_m-n_ = 0.04 μS. The transconductance of NPA-OTFTs are even slightly higher than in conventional ambipolar OTFTs. In contrast, the transconductance obtained from coplanar split-gate architectures is three times smaller. A comparison of the gate configurations, semiconductors, transistor architectures, and experimental application demonstrations is provided in Table 1. Avoiding the gap, the non-planar configuration offers the highest on-current and transconductance.

### Impact of the gaps on the transistor performance

**Figure 3:**
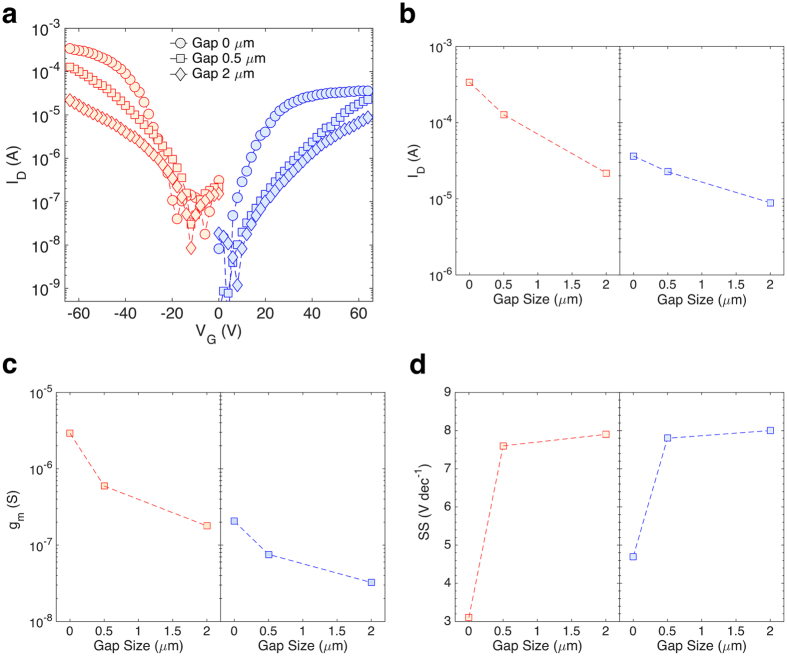
Impact of the gap on the transistors performance. (**a**) Measured transfer characteristics as a function of the gap size. |V_D_| = 60 V, and |V_side_| = 60 V. (**b**) Maximum on-current (viz. at |V_G_| = 65 V) as a function of the gap size when the transistor is operated as p-type (left panel) or n-type (right panel). (**c**) Maximum transconductance as a function of the gap size. (**d**) Maximum subthreshold slope as a function of the gap size.

In order to further assess the impact of the gap on the performance of ambipolar OTFTs, we intentionally fabricated NPA-OTFTs with a gap between the gate and the side gate (Supplementary Fig. 4). Figure 3a shows the measured transfer characteristics of NPA-OTFTs with 2 μm size gap, 0.5 μm size gap, and without gap. Figure 3b–d show the main transistor parameters, namely the maximum on-current, the transconductance, and the subthreshold slope, as a function of the gap size. The best performances are obtained when using NPA-OTFTs without gap. The measurements confirm that the transistor performances are dramatically affected by the gap size. By increasing the gap size the on-current and the transconductance become smaller and the subthreshold slope increases. More in detail, when the NPA-OTFTs is operated in p-type mode, the on-current (Fig. 3b, left panel) decreases by a factor of 3 and 15 when the gap size is 0.5 μm and 2 μm, respectively. Analogously, the maximum transconductance (Fig. 3c, left panel) is 2.97 μS in NPA-OTFTs without the gap, while it lowers to 0.54 μS and 0.11 μS when the gap size is 0.5 μm and 2 μm, respectively. Therefore, the transconductance is reduced by a factor of 25. In addition, the subthreshold slope (Fig. 3d, left panel) increases from 3 V/dec up to 7.6 V/dec by increasing the gap size. In the case of n-type operation, the on-current (Fig. 3b, right panel) decreases by a factor of 1.4 and 4 when the gap size is 0.5 μm and 2 μm, respectively, while the transconductance (Fig. 3c, right panel) reduces by a factor of 7. On the other hand, the subthreshold slope (Fig. 3d, right panel) increases from 4.7 V/dec to 8 V/dec when the gap size is 0.5 μm and 2 μm.

### 2D numerical simulations

To gain more insight on the key physical, material and geometrical parameters of the non-planar ambipolar OTFTs we reproduced the measurements with numerical simulations (Fig. 2a, full lines). The continuity, Poisson, and drift-diffusion transport equations are solved on a two-dimensional (2D) grid. Charge flow at the metal–semiconductor interface is calculated with the thermionic field emission equations accounting for the actual 2D energy barriers, and electric field distributions at the interface^37^,^38^. The simulation input parameters are given in Supplementary Table 1. In reproducing the measurements with 2D numerical simulations, we estimated the gold work function to be 4.7 eV, which is in good agreement with the 4.5–5.5 eV energy range^39^,^40^,^41^. Consequently, the charge injection barriers to electrons and holes were estimated to be approximately Φ_Be_ = 0.66 eV and Φ_Bh_ = 0.90 eV. The density of states (DOS) of the PDPP3T semiconductor is calculated by fitting the measurements over the whole range of applied voltages. We found that both the electron and hole DOS can be well approximated by the sum of two Gaussian functions defined by the total density of tail and deep states and disorder energy width. The hole and electron DOS are shown in the Supplementary Fig. 5. The DOS parameters show that the total number of LUMO and HOMO states is similar for both electrons and holes, while the energetic disorder (σ) is larger for electrons (σ_e_ = 90 meV and σ_h_ = 60 meV). This suggests that in PDPP3T the hole transport is easier than the electron transport. Dipoles due to the ODPA treatment are also included by means of surface charges at the insulator-semiconductor interface (N_is_ = 2 × 10^11 ^cm^−2^). ODPA is an alkane phosphonic acid-based SAM with positive charges facing the semiconductor^11^,^42^. This explains the different hole and electron on-voltages (V_on,h_ ≈ −20 V, V_on,e_ ≈ 0 V) obtained from the transfer characteristics shown in Fig. 2a.

**Figure 4:**
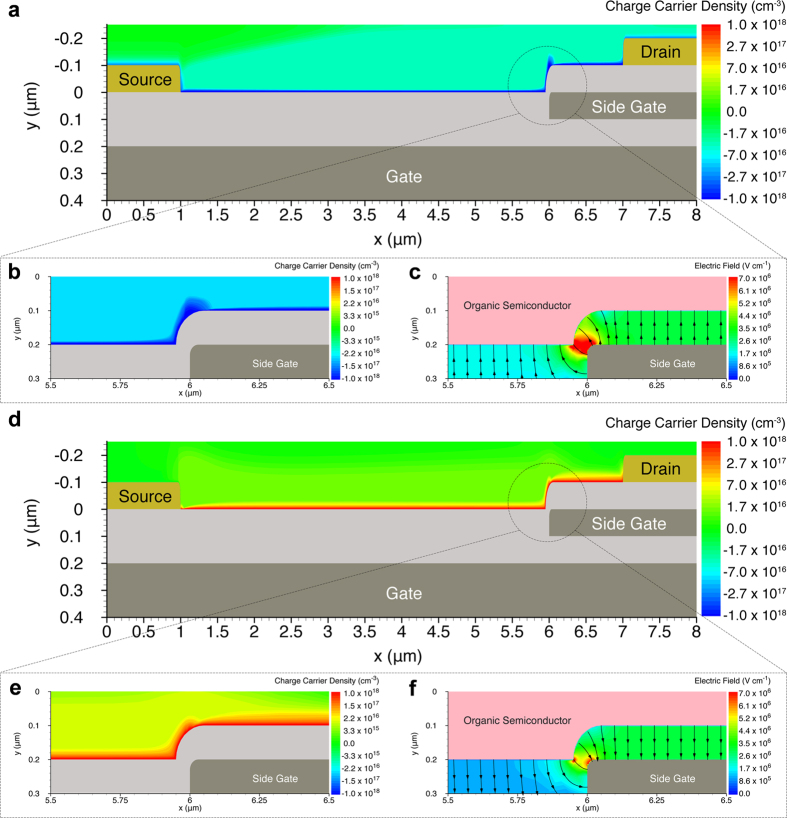
Operation of non-planar ambipolar transistors. 2D numerical simulations. The applied voltages are |V_G_| = 50 V, |V_D_| = 30 V, V_S_ = 0 V, |V_side_| = 60 V. Physical and geometrical parameters are given in the Supplementary Fig. 3 and the SEM images in the Supplementary Fig. 4, respectively. (**a**) N-type operation. Electron concentration into the organic semiconductor. (**b**) Zoomed image of the electron concentration accumulated at the side gate edge. (**c**) Zoomed image of the 2D distribution and streamline of the electric field at the side gate edge. (**d**) P-type operation. Hole concentration into the organic semiconductor. (**e**) Zoomed image of the hole concentration accumulated at the side gate edge. (**f**) Zoomed image of the 2D distribution and streamline of the electric field at the side gate edge.

The simulated transistor geometries are derived from the SEM cross-sections shown in the Supplementary Fig. 4. The charge concentration in the PDPP3T semiconductor and the electric field distribution in the gate insulator are shown when the NPA-OTFT without gap operates as n-type (Fig. 4a–c) or p-type transistor (Fig. 4d–f). In both cases a continuous charge accumulation is obtained along the whole channel (Fig. 4a,d). Figure 4b,e show a zoom of the charge concentration at the edge of the side gate. The side gate is deposited on top of the gate insulator and hence the semiconductor is spin-coated on a non-planar structure. Despite the non-planar structure of the transistor, full accumulation is attained also at the edge of the side gate. The charge concentration at the edge of the side gate is larger than the channel concentration owing to the 2D electric field distribution due to the non-planar geometry, as shown in Fig. 4c,f. Moreover, the charge concentration accumulated by side gate is larger than the charge concentration accumulated by the gate region because |V_side_| > |V_G_| and the side gate insulator thickness is the half of the main gate. This explains the higher on-current and transconductance obtained for NPA-OTFTs compared with conventional ambipolar OTFTs fabricated in the same technology (Supplementary Fig. 3).

**Figure 5:**
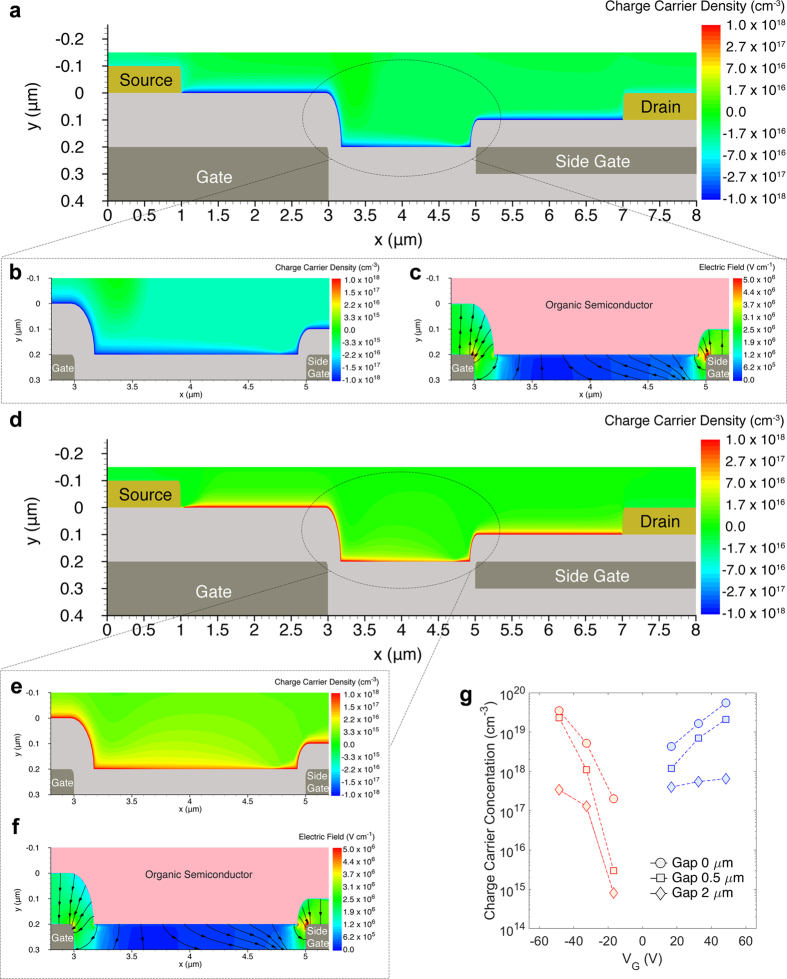
Operation of non-planar ambipolar transistors with gap. 2D numerical simulations. The applied voltages are |V_G_| = 50 V, |V_D_| = 30 V, V_S_ = 0 V, |V_side_| = 60 V. Physical and geometrical parameters are given in the Supplementary Figs. 3 and 4, respectively. (**a**) N-type operation. Electron concentration into the organic semiconductor. (**b**) Zoomed image of the electron concentration accumulated in the gap region. (**c**) Zoomed image of the 2D distribution of the electric field in the gap region. (**d**) P-type operation. Hole concentration into the organic semiconductor. (**e**) Zoomed image of the hole concentration accumulated in the gap region. (**f**) Zoomed image of the 2D distribution of the electric field in the gap region. (**g**) The charge concentration at the insulator/semiconductor interface in the middle of the gaps as a function of VG for NPA-OTFTs with different gap size.

Figure 5 shows the charge concentration and the electric field distribution when the NPA-OTFT with 2 μm gap is operated as n-type (Fig. 5a–c) or p-type transistor (Fig. 5d–f). The electron and hole concentration are shown in Fig. 5a,d, respectively. The charge carriers are accumulated in correspondence of the gate and the side gate and, owing to the lateral fringing of the electric field (Fig. 5c,f), good charge accumulation is also attained at both sides of the gap region. Despite the similar electric field distribution shown in Fig. 5c,f, Fig. 5b shows that in the case of n-type operation electrons are well-confined and almost completely accumulated in the gap region. In contrast, Fig. 5e shows that in the case of p-type operation holes are weakly accumulated and poorly confined in the central region of the gap. The smaller hole accumulation is inherently due to the alkane-based SAM dipoles orientation: the positive charges of the SAM dipole face the PDPP3T semiconductor, giving rise to a more favourable electron accumulation. Therefore, in the case of p-type operation the parasitic resistance due to the gap is large and about 50% of V_D_ drops on the gap region. This has also a negative impact on the charge injection which strongly depends on the drain voltage in a bottom-gate bottom-contact structure^43^. The larger injection barrier and the reduced V_D_ result in an inefficient charge injection, and hence a hole depletion close to the source contact is readily visible in Fig. 5d. This reveals that the gap has a double detrimental effect on the transistor performance since it causes increase in parasitic series resistance and reduced charge injection. The detrimental effect of the gap is also strictly related to the SAM treatment. For example, fluorinated-alkyl SAMs would result in negative interface charges and thus hole accumulation. In addition, the SAM dipole strength can vary several orders of magnitude and, depending on the process conditions, it gives rise to interface charges in the range 10^11^–10^13 ^cm^−2^ 42,44.

**Figure 6:**
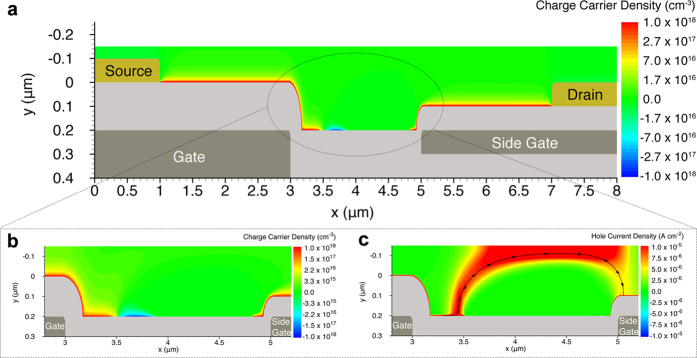
Impact of the SAM treatment and charge carrier concentration in the gate-gap region on the transistor operation. The interface charges due to the dipoles of the SAM are deliberately increased from 2 × 10^11 ^cm^−2^ (as in Figs 4 and 5) to 7 × 10^11 ^cm^−2^. (**a**) Hole and electron concentration into the organic semiconductor. (**b**) Zoomed image of the hole and electron concentration into the gap region. (**c**) Zoomed image of the hole current density distribution into the gap region. Holes transport in the bulk of the semiconductor and the drain current is lower than 10^−12^ A.

To further investigate the impact of the SAM on the gaps, we deliberately increase the interface charges from 2 × 10^11 ^cm^−2^ to 7 × 10^11 ^cm^−2^. Figure 6 shows the charge carrier concentration and the hole current density when the transistor is operated as p-type. Surprisingly, we found that p-type operation is prevented. The weak electric field in the central part of the gap is not large enough to compensate the electron accumulation. Figure 6b shows that a reversed biased p-n junction is formed at the left side of the gap, while the right side is fully depleted. Although the transistor is operated in the on-state (V_G_ = −30 V, V_D_ = −30 V, V_side_ = −60 V), the charge carriers transport in the bulk of the semiconductor and the drain current is lower than 10^−12^ A (Fig. 6c). Therefore, we can conclude that the presence of the gap in split-gate ambipolar transistors not only limits the performances but may also result in non-functional devices when the fabrication process is not properly optimized.

### High-performance complementary inverters

**Figure 7:**
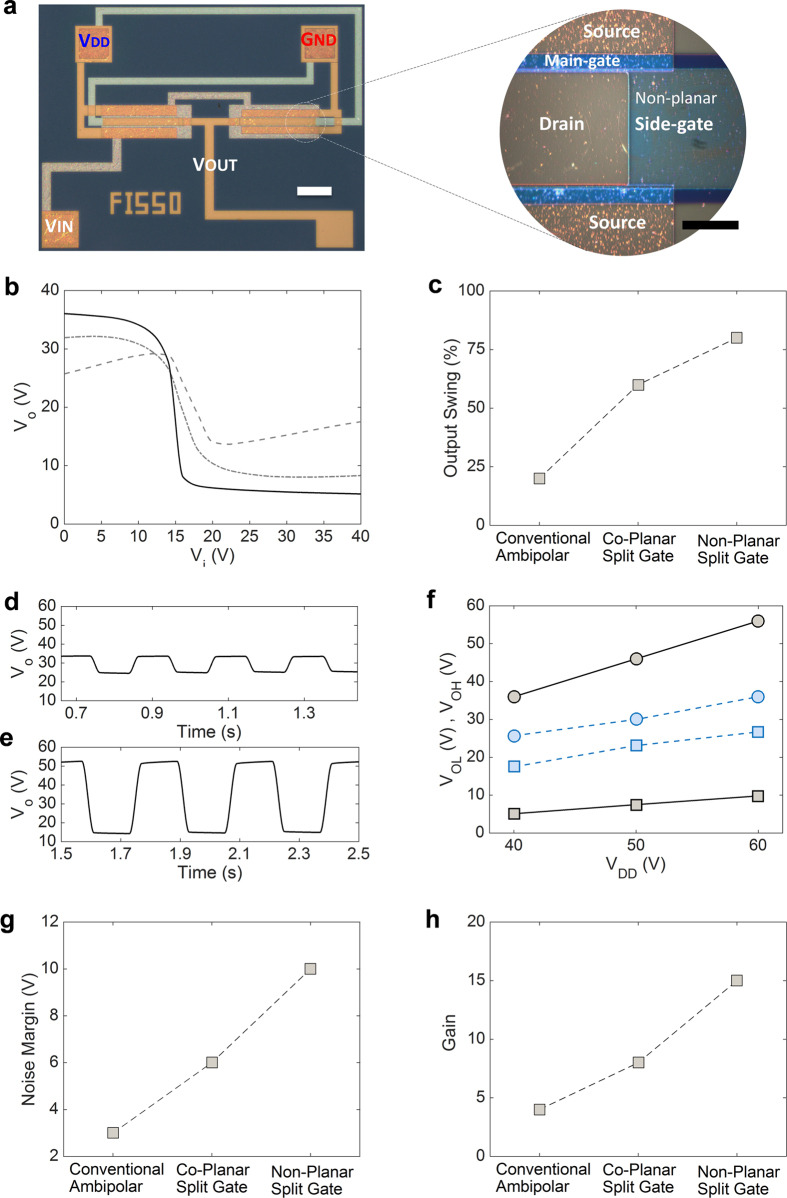
Impact of the transistor structure on the inverters performance. (**a**) Optical microscopy image of non-planar ambipolar inverter, scale bar is 200 μm (white) and zoom of the channel region, scale bar is 20 μm (black). (**b**) Measured inverter characteristics (V_o_-V_i_) of conventional (dashed line), co-planar split gate with gap (dotted line), and non-planar without gap (full line) ambipolar organic transistors. (**c**) Output swing as a function the transistor structure. Output voltage response of (**d**) conventional ambipolar and (**e**) non-planar ambipolar inverters at V_DD_ = 60 V. (**f**) Low (squares, V_OL_) and high (circles, V_OH_) output voltage of conventional (dashed lines) and non-planar without gap (full lines) ambipolar inverters as a function of the supply voltage V_DD_ (G_ND_ = 0 V). (**g**) Noise margin and (**h**) gain as a function of the transistor structure.

Next, we fabricated the inverters with various transistor structures. Figure 7a shows the optical image of NPA-OTFT-based inverter and the zoomed image of the channel region to confirm the absence of gate-gap. Typical transfer characteristics of complementary inverters based on conventional ambipolar OTFTs (dashed line), split-gate ambipolar OTFTs (dot-dashed line) and NPA-OTFT (full line) are shown in Fig. 7b. All the inverters are fabricated in the same technology. The main figures of merit of the inverters, viz. output swing, noise margin, and gain, are displayed in Fig. 7c–h, respectively. The inverters based on the proposed NPA-OTFTs show the best characteristics. The maximum gain is 15, noise margin is 10 V, and the output swing is larger than 75% of V_DD._ As shown in Fig. 7f the low and high output voltages (V_OL_ and V_OH_) measured in NPA-OTFTs based inverters are very close to V_DD_ and G_ND_ showing a three-fold improvement of the output swing (V_OH_-V_OL_) with respect to inverters fabricated with conventional ambipolar transistors. Moreover, in NPA-OTFTs inverters the output swing increases with the supply voltage since the side gates of the NPA-OTFTs operated in n-type and p-type mode are connected to V_DD_ and G_ND_, respectively. Hence, with respect to inverters based on conventional ambipolar OTFTs, the overall performances are improved by a factor of 3. The low performance of the conventional ambipolar inverters OTFTs in terms of noise margin and output swing are inherently related to the Z-shaped characteristic (dashed line Fig. 7b) due to the ambipolar conduction. Furthermore, the experimental results show that modest noise margin – which is crucial for the development of robust electronics – of split-gate inverters is due to the gap between the gate and the side gate, and this is in agreement with other previous works^33^.

### Reconfigurable complementary logic gates

**Figure 8:**
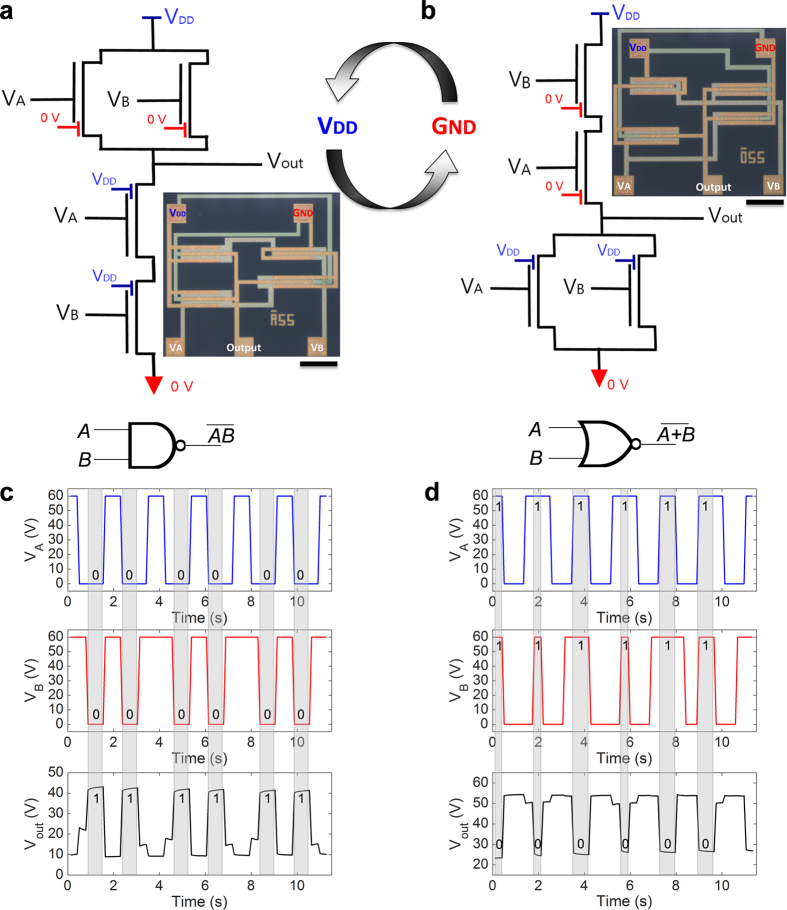
Electrically reconfigurable complementary logic gates. (**a**) Schematic, symbol, and top-view image of a complementary logic NAND, scale bar is 400 μm. (**b**) Schematic, symbol, and top-view image of a complementary logic NOR, scale bar is 400 μm. (c) Inputs and measured output of the NAND circuits. (**d**) Inputs and measured output of the NOR circuits. The NAND circuits can be electrically reconfigured in NOR circuits by simply swapping V_DD_ and G_ND_ = 0V.

NAND and NOR circuits are key components for digital integrated circuits. A logic operation of any complexity can be built using NAND or NOR gates only^45^ and hence NAND and NOR gates are universal gates. In addition, the efficiency in terms of function complexity per transistor count can be improved when both NAND and NOR are available. NAND and NOR logic circuits based on NPA-OTFTs are shown in Fig. 8a,b, respectively. P- and n-type transistor operations are obtained by simply connecting the side gate to G_ND_ = 0 V and V_DD_, respectively. This prevents the undesired charge injection from the drain electrode. Since the polarity of the transistors depends on the side gate voltage, a NAND circuit can be electrically reconfigured into a NOR circuit and vice versa. Electrically reconfigurable gates find relevant application for the development of programmable logic circuits, field-programmable gate arrays, and microprocessors^9^,^46^,^47^. Moreover, the availability of both NAND and NOR gates provides a more efficient implementation in terms of number of logic gates and mapping functions. By the way of example, in a NAND-only design the NOR function can be obtained with four NANDs. Figure 8c,d show the NAND and NOR operation. Inputs A and B are applied as a function of time and the switching characteristics of NAND and NOR gates showed that a corresponding output voltages changed properly. This is the first demonstration, at least to the authors’ knowledge, of complementary logic circuits fabricated with ambipolar organic transistors.

In summary, non-planar ambipolar OTFTs offer the simple fabrication of ambipolar organic reconfigurable complementary logic circuits. NPA-OTFTs can electrically control the transistor polarity providing orders of magnitude superior performances with respect to state-of art coplanar multiple gate ambipolar transistors. 2D numerical simulations provide insight on the transistor operation and the key design and material parameters. The simulations reveal that, in contrast to the NPA-OTFTs, coplanar multiple gate ambipolar transistors have to be carefully optimized and the insulator-semiconductor interface plays a key role for the proper transistor operation. This is critical for the development of practical applications. Electrically reconfigurable complementary logic gates based on NPA-OTFTs are experimentally demonstrated for the first time. This opens up new opportunities for simple and low-cost fabrication of large-area complementary logic organic circuits.

## Methods

### Devices fabrication

Bottom gate/bottom contact ambipolar TFTs were fabricated on having 300 nm SiO_2_ on Si substrate. Bottom gate electrodes (aluminum, 200 nm) and middle gate electrodes (aluminum, 100 nm) were deposited using an e-beam evaporator and were patterned using the dry metal etching method. Two gate dielectric layers, aluminum oxide (100 nm), were deposited on top of patterned bottom and middle aluminum gate electrodes using the atomic layer deposition method. The source/drain electrodes (Au, 100 nm) were deposited on top of the aluminum oxide using e-beam evaporation and lift-off lithography. Inductively Coupled Plasma (ICP) etching was used for oxide etching to form via-holes from bottom and middle gate electrodes to source/drain electrodes. All the transistors have the same channel lengths and widths equal to L = 6 μm and W = 810 μm, respectively. For the surface treatment on top of channel region, the samples were dipped in a solution of 10 mM of octadecylphosphonic acid (ODPA) in Isopropyl alcohol (IPA) for 3–5 days after being exposed to UV-ozone for 15 min. Then, PDPP3T, from Solarmer, was dissolved in 1, 2-dichlorobenzene (ODCB) to obtain 13 mg mL^−1^ and spin-coated. The samples were vacuum-dried at 100 °C for longer than 12 hours to remove any residue of the solvent and then annealed at 150 °C for 1 hour in ultra-high vacuum (<10^−6^ torr).

### Electrical characterizations

All devices were measured in a vacuum probe station (Keithley 4200-SCS) and LCR meter (E4980A). The transconductance was extracted in the saturation regime using the following equations: g_m_ = dI_D_/dV_G_.

### Two-dimensional numerical simulations

The coupled drift-diffusion, Poisson, and current continuity equations are solved together^33^,^37^,^38^. The electron and hole DOS are well approximated by the sum of two Gaussian functions. The DOS is shown in Supplementary Fig. 5 and the DOS parameters are listed in the Supplementary Table S1. The simulation parameters are the following: relative permittivity of semiconductor ε_rs_ = 3, relative permittivity of insulator ε_ri_ = 9, highest occupied molecular orbital (HOMO) energy level E_HOMO_ = 5.60 eV, lowest unoccupied molecular orbital (LUMO) energy level E_LUMO_ = 4.04 eV, holes mobility μ_h_ = 0.5 cm^2 ^V^−1^ s^−1^, electrons mobility μ_e_ = 0.1 cm^2 ^V^−1 ^s^−1^, gold electrodes work function Φ_Au_ = 4.7 eV (the hole and electron energy barrier at the source/drain metal-semiconductor are Φ_Bh_ = 0.9 eV and Φ_Be_ = 0.66, respectively), Schottky barrier lowering ΔΦ_B_ = e [e E/(4 π ε_0_ ε_rs_)]^1/2^, where e is the elementary charge, E is the electric field, and ε_0_ is the vacuum permittivity.

## Additional Information

**How to cite this article**: Yoo, H. *et al*. Reconfigurable Complementary Logic Circuits with Ambipolar Organic Transistors. *Sci. Rep.* **6**, 35585; doi: 10.1038/srep35585 (2016).

## Supplementary Material

Supplementary Information

## Figures and Tables

**Table 1:** Comparison of the transistor structures, (L/W) normalized on-current and transconductance, and application demonstrations.

Gate structure	Semiconductor	Transistor architecture	# of gaps	Gap size [μm]	(L/W) × I_ON-p_[nA]	(L/W) × I_ON-n_[nA]	(L/W) × g_m-p_[pS]	(L/W) × g_m-n_ [pS]	Experimental application	Ref.
Coplanar split-gate	PCDTBT:PC_70_BM blend	Bottom-gate top-contact	1	4	41	3	267	258	—	[31]
Coplanar split-gate	F8BT	Bottom-gate top-contact	1	4	11	7	0.8	0.2	Light-emitting transistors	[32]
Coplanar tri-gate	PDPPTPT	Bottom-gate bottom-contact	2	1.5	4	4	11	18	—	[33]
Coplanar split-gate	PDPP-TT-T	Bottom-gate bottom-contact	1	1	320	32	810	405	CMOS inverter	[34]
Non-planar split-gate	PDPP3T	Bottom-gate bottom-contact	0	0	2960	296	12400	1400	Reconfigurable logic gates	This work

## References

[b1] Gelinck G. H. . Flexible active-matrix displays and shift registers based on solution-processed organic transistors. Nat. Mater. 3, 106–110 (2004). 14743215 10.1038/nmat1061

[b2] Sekitani T. . A large-area wireless power-transmission sheet using printed organic transistors and plastic MEMS switches. Nat. Mater. 6, 413–417 (2007). 17468763 10.1038/nmat1903

[b3] Schwartz G. . Flexible polymer transistors with high pressure sensitivity for application in electronic skin and health monitoring. Nat. Commun. 4, 1859 (2013). 23673644 10.1038/ncomms2832

[b4] Kaltenbrunner M. . An ultra-lightweight design for imperceptible plastic electronics. Nature 499, 458–463 (2013). 23887430 10.1038/nature12314

[b5] Jung S., Sou A., Gili E. & Sirringhaus H. Inkjet-printed resistors with a wide resistance range for printed read-only memory applications. Org. Electron. 14, 699–702 (2013).

[b6] Myny K. . Unipolar organic transistor circuits made robust by dual-gate technology. IEEE J. Solid-State Circuits 46, 1223–1230 (2011).

[b7] Klauk H., Zschieschang U., Pflaum J. & Halik M. Ultralow-power organic complementary circuits. Nature 445, 745–748 (2007). 17301788 10.1038/nature05533

[b8] Torsi L., Magliulo M., Manoli K. & Palazzo G. Organic field-effect transistor sensors: a tutorial review. Chem. Soc. Rev. 42, 8612–8628 (2013). 24018860 10.1039/c3cs60127g

[b9] Sou A. . Programmable logic circuits for functional integrated smart plastic systems. Org. Electron. 15, 3111–3119 (2014).

[b10] Baeg K.-J. . Flexible Complementary Logic Gates Using Inkjet-Printed Polymer Field-Effect Transistors. IEEE Electron Device Lett. 34, 126–128 (2013).

[b11] Zhang Z. . Direct Patterning of Self-Assembled Monolayers by Stamp Printing Method and Applications in High Performance Organic Field-Effect Transistors and Complementary Inverters. Adv. Funct. Mater. 25, 6112–6121 (2015).

[b12] Kraft U. . Flexible low-voltage organic complementary circuits: Finding the optimum combination of semiconductors and monolayer gate dielectrics. Adv. Mater. 27, 207–214 (2015). 25330764 10.1002/adma.201403481

[b13] Yoo H. . Self-assembled, millimeter-sized TIPS-pentacene spherulites grown on partially crosslinked polymer gate dielectric. Adv. Funct. Mater. 25, 3658–3665 (2015).

[b14] James D. T. . Thin-Film Morphology of Inkjet- Printed Single-Droplet Organic Transistors Using Polarized Raman Spectroscopy: Effect of Blending TIPS- Pentacene with Insulating Polymer. ACS Nano 5, 9824–9835 (2011). 22032725 10.1021/nn203397m

[b15] Shin N. . Vertically Segregated Structure and Properties of Small Molecule-Polymer Blend Semiconductors for Organic Thin-Film Transistors. Adv. Funct. Mater. 23, 366–376 (2013).

[b16] Cho J. . Facile and Fine Polarity-Tuning of Polymeric Semiconductors: The Effects of Nitrile Groups on Polymer-Polymer Blend Systems. Adv. Electron. Mater. 2 (2016).

[b17] Benor A. & Knipp D. Contact effects in organic thin film transistors with printed electrodes. Org. Electron. 9, 209–219 (2008).

[b18] Timmreck R., Olthof S., Leo K. & Riede M. K. Highly doped layers as efficient electron-hole recombination contacts for tandem organic solar cells. J. Appl. Phys. 108 (2010).

[b19] Lee J. . Ionic self-assembled monolayer for low contact resistance in inkjet-printed coplanar structure organic thin-film transistors. Org. Electron. 15, 2021–2026 (2014).

[b20] Xu Y. . Regulating charge injection in ambipolar organic field-effect transistors by mixed self-assembled monolayers. ACS Appl. Mater. Interfaces 6, 14493–14499 (2014). 25093699 10.1021/am5037862

[b21] Bock C. . Improved morphology and charge carrier injection in pentacene field-effect transistors with thiol-treated electrodes. J. Appl. Phys. 100, 114517 (2006).

[b22] Kwon J., Kyung S., Yoon S., Kim J.-J. & Jung S. Solution-Processed Vertically Stacked Complementary Organic Circuits with Inkjet-Printed Routing. Adv. Sci. 3 (2016). 10.1002/advs.201500439 PMC5067658 27812468

[b23] Chung S., Kim S. O., Kwon S.-K., Lee C. & Hong Y. All-Inkjet-Printed Organic Thin-Film Transistor Inverter on Flexible Plastic Substrate. IEEE Electron Device Lett. 32, 1134–1136 (2011).

[b24] Myny K. . A thin-film microprocessor with inkjet print-programmable memory. Sci. Rep. 4, 7398 (2014). 25492120 10.1038/srep07398 PMC4261169

[b25] Bisri S. Z., Piliego C., Gao J. & Loi M. A. Outlook and emerging semiconducting materials for ambipolar transistors. Adv. Mater. 26, 1176–1199 (2014). 24591008 10.1002/adma.201304280

[b26] Lei T., Dou J. H., Cao X. Y., Wang J. Y. & Pei J. A BDOPV-based donor-acceptor polymer for high-performance n-type and oxygen-doped ambipolar field-effect transistors. Adv. Mater. 25, 6589–6593 (2013). 23970421 10.1002/adma.201302278

[b27] Deng Y. . Black Phosphorus-Monolayer MoS2 van der Waals Heterojunction P-N Diode. ACS Nano 8, 8292–8299 (2014). 25019534 10.1021/nn5027388

[b28] Buscema M., Groenendijk D. J., Steele G. a., van der Zant H. S. J. & Castellanos-Gomez A. Photovoltaic effect in few-layer black phosphorus PN junctions defined by local electrostatic gating. Nat. Commun. 5, 4651 (2016). 10.1038/ncomms5651 25164986

[b29] Maruyama K. . Ambipolar light-emitting organic single-crystal transistors with a grating resonator. Sci. Rep. 5, 10221 (2015). 25959455 10.1038/srep10221 PMC4426699

[b30] Zhou X. . Balanced ambipolar organic thin-film transistors operated under ambient conditions: Role of the donor moiety in BDOPV-based conjugated copolymers. Chem. Mater. 27, 1815–1820 (2015).

[b31] Hsu B. B. Y. . Split-gate organic field effect transistors: Control over charge injection and transport. Adv. Mater. 22, 4649–4653 (2010). 20839245 10.1002/adma.201001509

[b32] Hsu B. B. Y. . Control of efficiency, brightness, and recombination zone in light-emitting field effect transistors. Adv. Mater. 24, 1171–1175 (2012). 22278999 10.1002/adma.201103513

[b33] Torricelli F. . Ambipolar Organic Tri-Gate Transistor for Low-Power Complementary Electronics. Adv. Mater. 28, 284–290 (2016). 26573767 10.1002/adma.201503414

[b34] Yoo H. . Asymmetric Split-Gate Ambipolar Transistor and Its Circuit Application to Complementary Inverter. Adv. Mater. Technol. 1 (2016).

[b35] Bijleveld J. C. . Poly(diketopyrrolopyrrole−terthiophene) for Ambipolar Logic and Photovoltaics. J. Am. Chem. Soc. 131, 16616–16617 (2009). 19886605 10.1021/ja907506r

[b36] Roelofs W. S. C. . Fast ambipolar integrated circuits with poly(diketopyrrolopyrrole- terthiophene). Appl. Phys. Lett. 98, 96–98 (2011).

[b37] Torricelli F., Colalongo L., Raiteri D., Kovács-Vajna Z. M. & Cantatore E. Ultra-high gain diffusion-driven organic transistor. Nat. Commun. 7, 10550 (2016). 26829567 10.1038/ncomms10550 PMC4740436

[b38] Lenz T. . Downscaling and Charge Transport in Nanostructured Ferroelectric Memory Diodes Fabricated by Solution Micromolding. Adv. Funct. Mater. 26, 5111–5119 (2016).

[b39] Chen Z. . High-Performance Ambipolar Diketopyrrolopyrrole-Thieno[3,2- *b* ]thiophene Copolymer Field-Effect Transistors with Balanced Hole and Electron Mobilities. Adv. Mater. 24, 647–652 (2012). 21997483 10.1002/adma.201102786

[b40] Khim D. . Control of Ambipolar and Unipolar Transport in Organic Transistors by Selective Inkjet-Printed Chemical Doping for High Performance Complementary Circuits. Adv. Funct. Mater. 24, 6252–6261 (2014).

[b41] Braun S., Salaneck W. R. & Fahlman M. Energy-level alignment at organic/metal and organic/organic interfaces. Adv. Mater. 21, 1450–1472 (2009).

[b42] Pernstich K. P. . Threshold Voltage Shift in Organic Field Effect Transistors by Dipole-Monolayers on the Gate Insulator. J. Appl. Phys. 96, 6431 (2004)

[b43] Torricelli F., Ghittorelli M., Colalongo L. & Kovacs-Vajna Z. M. Single-transistor method for the extraction of the contact and channel resistances in organic field-effect transistors. Appl. Phys. Lett. 104, 1–5 (2014).

[b44] Kobayashi S. . Control of carrier density by self-assembled monolayers in organic field-effect transistors. Nat. Mater. 3, 317–322 (2004). 15064756 10.1038/nmat1105

[b45] Myny K. . An 8-Bit, 40-Instructions-Per-Second Organic Microprocessor on Plastic Foil. IEEE J. Solid-State Circuits 47, 284–291 (2012).

[b46] Ishida K. . User Customizable Logic Paper (UCLP) with Sea-of Transmission-Gates (SOTG) of 2-V organic CMOS and ink-jet printed interconnects. IEEE J. Solid-State Circuits 46, 285–292 (2011).

[b47] Li X. . Programmable polymer light emitting transistors with ferroelectric polarization-enhanced channel current and light emission. Org. Electron. 13, 1742–1749 (2012).

