# Supplementary material for: Reconfigurable Complementary Logic Circuits with Ambipolar Organic Transistors
Source: Sci Rep. 2016 Oct 20;6:35585. doi: 10.1038/srep35585 (PMC5071863; doi:10.1038/srep35585)
Supplement: Supplementary Information [file srep35585-s1.pdf]

Supplementary Information

Reconfigurable Complementary Logic Circuits with Ambipolar Organic Transistors

Hocheon Yoo[1], Matteo Ghittorelli*[2], Edsger C. P. Smits[3], Gerwin H. Gelinck[3], Han-Koo Lee[4], Fabrizio Torricelli†[2], and Jae-Joon Kim‡[1]*

[1] Department of Creative IT Engineering, Pohang University of Science and Technology (POSTECH), Pohang 790-784, Korea

[2] Department of Information Engineering, Università degli Studi di Brescia, via Branze 38, 25123 Brescia, Italy

[3] Holst Centre, TNO-The Dutch Organization for Applied Scientific Research, High Tech Campus 31, 5656 AE Eindhoven, The Netherlands

[4] Pohang Accelerator Laboratory, Pohang 790-784, Korea

* H. Yoo and M. Ghittorelli contributed equally to this work

† Correspondence should be addressed to fabrizio.torricelli@unibs.it

‡ Correspondence should be addressed to jaejoon@postech.ac.kr

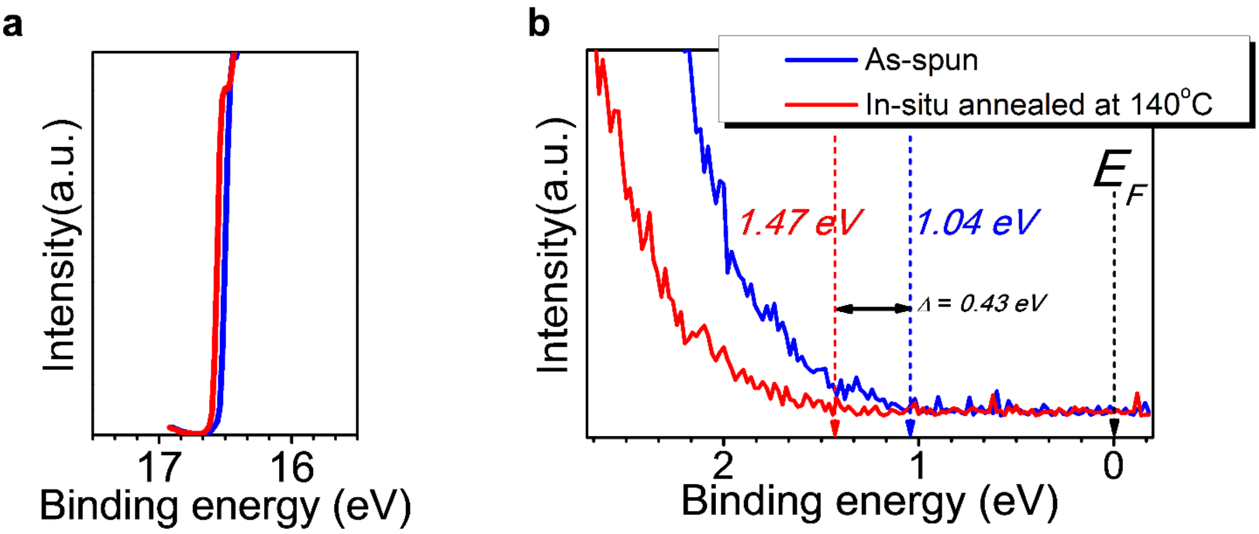

Supplementary Figure 1 | Ultraviolet photoelectron spectroscopy (UPS) data of the PDPP3T film showing the shift of HOMO with performing the vacuum annealing. (a) The secondary cut-off region and (b) the HOMO region. From UPS spectrum of PDPP3T, the shift of the HOMO and LUMO was observed. First, we employed values of 1.36, 5.17, and 3.61 eV for the band-gap, HOMO, and LUMO, respectively for PDPP3T in 1, 2-dichlorobenzene (ODCB) solution, which was reported by Janssen et al.[1] In Supplementary Figure 1, the film annealed in vacuum produced the shift-down of the HOMO as much as Δ = 0.43 eV while EF was almost pinned. Thus, the HOMO and LUMO are 5.60 and 4.04 eV, respectively in vacuumed state after in-situ annealing at 140 ℃. The change in energy band can be attributed to a removal of an interfacial oxygen layer during vacuum annealing period.[2–4] The rearrangement of energy-bands by thermal annealing resulted in the occurrence of electron carrier injection. Consequently, it enabled PDPP3T to operate in ambipolar mode as seen in Supplementary Figure 2.

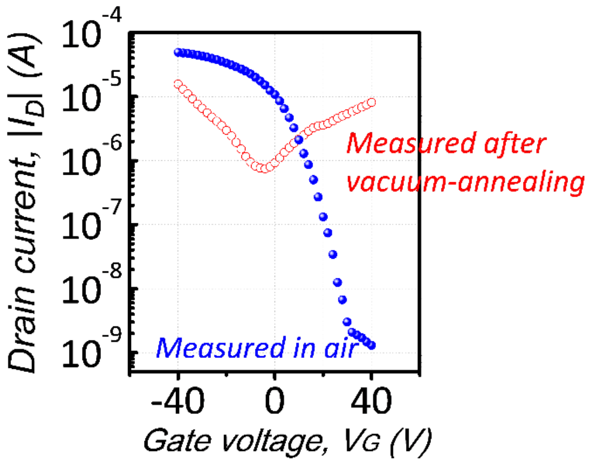

Supplementary Figure 2 | Electrical characteristics of PDPP3T TFTs when measured in air and vacuum after thermal annealing at 140 ℃. The device is biased at V_D = -30 V. The transistors channel length and width are L = 6 µm and W = 810 µm, respectively. The conventional single-gate ambipolar transistors are fabricated in the same technology of the non-planar ambipolar transistors.

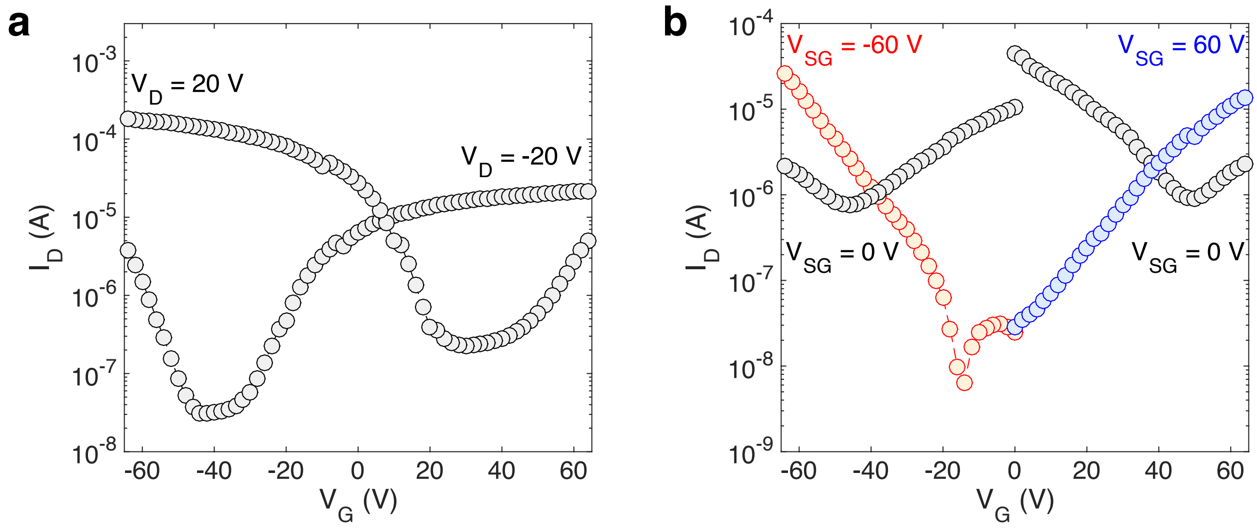

Supplementary Figure 3 | Electrical characteristics of ambipolar transistors. The transistors channel length and width are L = 6 µm and W = 810 µm, respectively. (a) Measured transfer characteristics (I_D-V_G) of conventional ambipolar transistors. (b) Measured I_D-V_G of split gate transistors with 1 µm gap. The transistors are fabricated in the same technology of the non-planar ambipolar transistors. Fabrication details are provided in the *Methods* section.

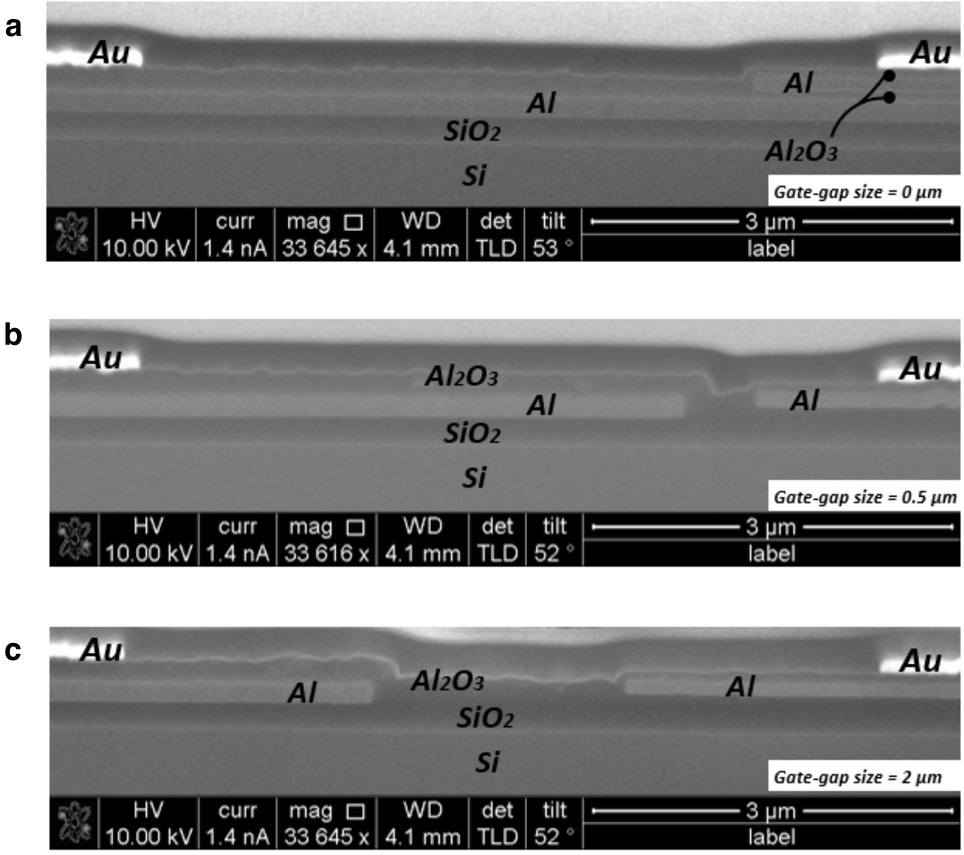

Supplementary Figure 4 | SEM cross-sections of non-planar ambipolar transistors. (a) Without gap. (b) Gap size is 0.5 µm. (c) Gap size is 2 µm.

Total density of HOMO tail states	N_{th} = 1.5x10^{20} cm^{-3}
Energy width of the HOMO tail-states distribution	σ_{th} = 0.06 eV
Total density of HOMO deep states	N_{dh} = 1.5x10^{19} cm^{-3}
Energy width of the HOMO deep-states distribution	σ_{dh} = 0.2 eV
Total density of LUMO tail states	N_{te} = 1.5x10^{20} cm^{-3}
Energy width of the LUMO tail-states distribution	σ_{te} = 0.09 eV
Total density of LUMO deep states	N_{de} = 4x10^{19} cm^{-3}
Energy width of the LUMO deep-states distribution	σ_{de} = 0.2 eV

Supplementary Table 1 | Physical parameters used as input data in the numerical simulations

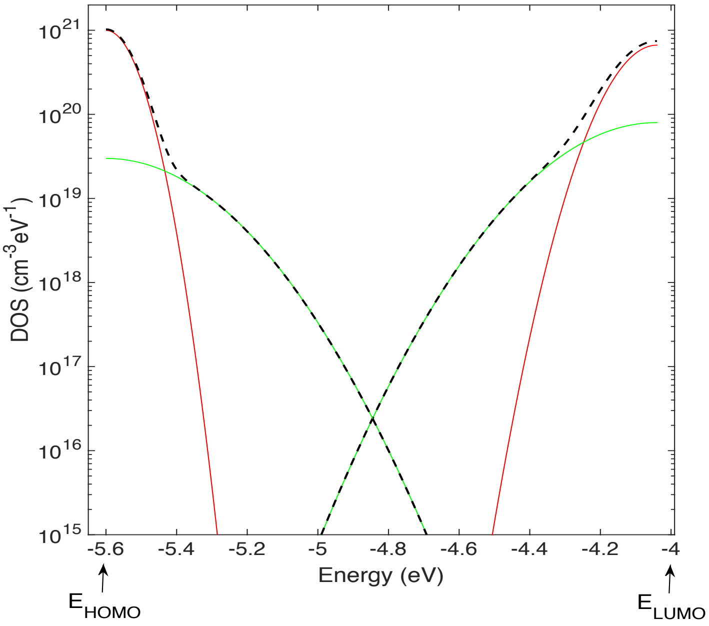

Supplementary Figure 5 | Electron and hole density of states. The dashed line is the overall DOS approximated by the sum of two Gaussian functions. The tail (red line) and deep (green line) states are also shown. The electron DOS parameters are the following. Total density of LUMO tail and deep states are N_{te} = 1.5×10^{20} cm^{-3} and N_{de} = 4×10^{19} cm^{-3}, respectively. The energy width of the LUMO tail and deep states are σ_{te} = 90 meV and σ_{de} = 200 meV, respectively. The hole DOS parameters are the following. Total density of HOMO tail and deep states are N_{th} = 1.5×10^{20} cm^{-3} and N_{dh} = 1.5×10^{19} cm^{-3}, respectively. The energy width of the HOMO tail and deep states are σ_{th} = 60 meV and σ_{dh} = 200 meV, respectively. The other simulation parameters are provided in the *Methods* section.

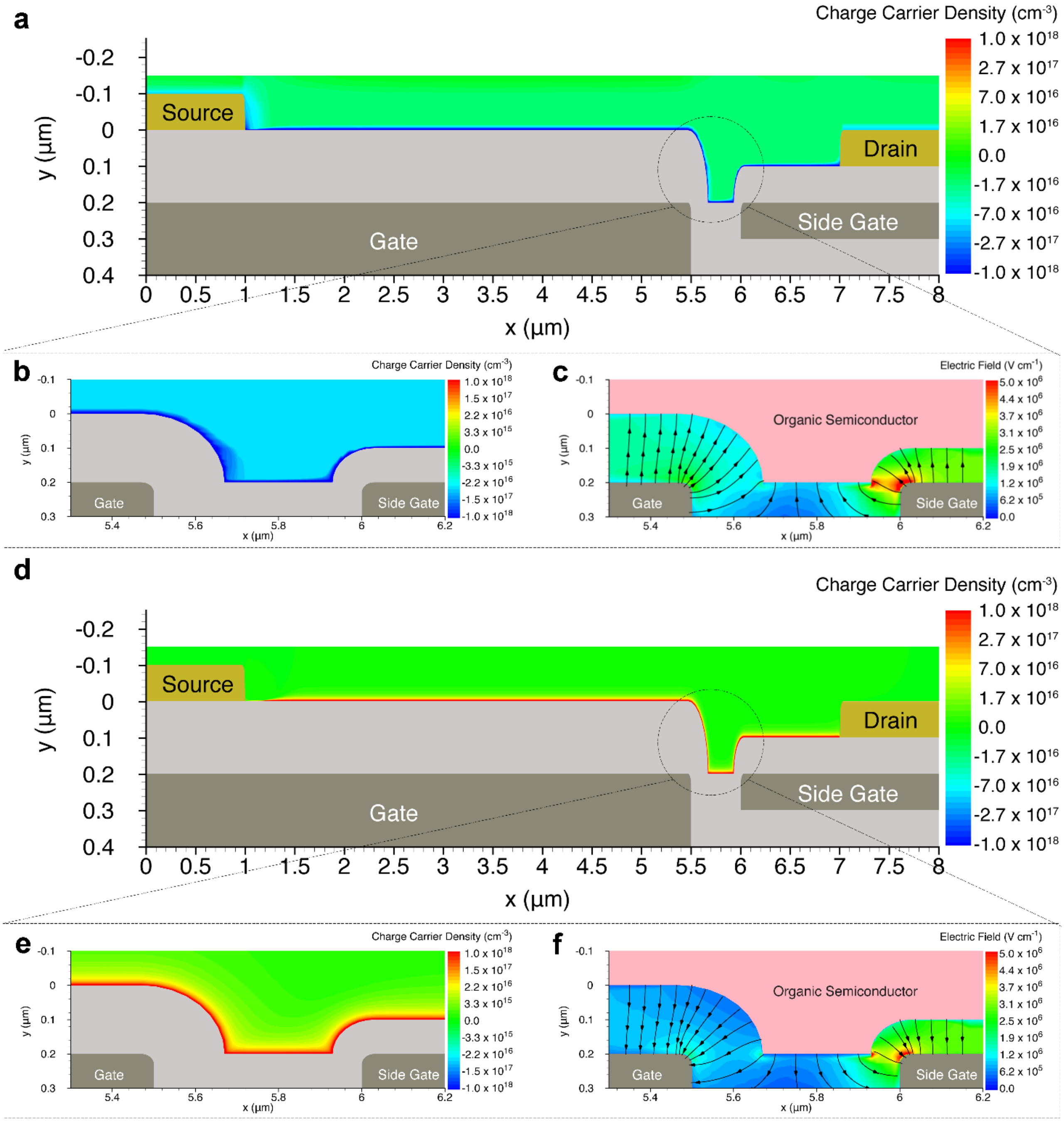

Supplementary Figure 6 | Operation of non-planar ambipolar transistors with 0.5 µm gap. 2D numerical simulations. The applied voltages are $|V_G|$ = 50 V, $|V_D|$ = 30 V, V_S = 0 V, $|V_{SG}|$ = 60 V. Physical and geometrical parameters are given in the Supplementary Figure. 3 and 4, respectively. (a) N-type operation. Electron concentration into the organic semiconductor. (b) Zoom of the electron concentration accumulated in the gap region. (c) Zoom of the 2D distribution of the electric field in the gap region. (d) P-type operation. Hole concentration into the organic semiconductor. (e) Zoom of the hole concentration accumulated in the gap region. (f) Zoom of the 2D distribution of the electric field in the gap region.

Supplementary References

[1] Bijleveld, J. C. *et al.* Poly(diketopyrrolopyrrole−terthiophene) for Ambipolar Logic and Photovoltaics. *J. Am. Chem. Soc.* **131,** 16616–16617 (2009).

[2] Nishi, T., Kanai, K., Ouchi, Y., Willis, M. R. & Seki, K. Evidence for the atmospheric p-type doping of titanyl phthalocyanine thin film by oxygen observed as the change of interfacial electronic structure. *Chem. Phys. Lett.* **414,** 479–482 (2005).

[3] Tanaka, Y., Kanai, K., Ouchi, Y. & Seki, K. Oxygen effect on the interfacial electronic structure of C60 film studied by ultraviolet photoelectron spectroscopy. *Chem. Phys. Lett.* **441,** 63–67 (2007).

[4] Di Pietro, R. & Sirringhaus, H. High resolution optical spectroscopy of air-induced electrical instabilities in n-type polymer semiconductors. *Adv. Mater.* **24,** 3367–3372 (2012).
